# Frailty: an in-depth qualitative study exploring the views of community care staff

**DOI:** 10.1186/s12877-019-1069-3

**Published:** 2019-02-19

**Authors:** J. F. Coker, M. E. Martin, R. M. Simpson, L. Lafortune

**Affiliations:** 1 Cambridge Institute of Public Health, School of Clinical Medicine, University of Cambridge, Cambridge, CB2 0SR UK; 2 Cambridgeshire and Peterborough NHS Foundation Trust, Elizabeth House, Fulbourn Hospital, Cambridge, CB21 5EF UK

**Keywords:** Frailty, Older adults, Community care, Healthcare professionals, Qualitative research

## Abstract

**Background:**

Frailty is seen across various health and social care settings. However, little is known about how healthcare professionals, particularly those who provide care for older adults living in the community view frailty. There is also a dearth of information about the extent to which a shared understanding of frailty exists across the various disciplines of care. Such an understanding is crucial across care professionals as it ensures consistent assessment of frailty and facilitates interdisciplinary working/collaboration which is a key component in the management of frailty. This study aimed to explore: (i) how community care staff from various specialties viewed frailty; (ii) whether they had a shared understanding; and (iii) how they assessed frailty in everyday practice.

**Methods:**

Semi-structured interviews were conducted with a purposive sample of 22 community care staff from seven specialties, namely: healthcare assistants, therapy assistants, psychiatric nurses, general nurses, occupational therapists, physiotherapists and social workers, recruited from four neighbourhood teams across Cambridgeshire, England. Interviews were analysed thematically.

**Results:**

There was a shared narrative among participants that frailty is an umbrella term that encompasses interacting physical, mental health and psychological, social, environmental, and economic factors. However, various specialities emphasised the role of specific facets of the frailty umbrella. The assessment and management of frailty was said to require a holistic approach facilitated by interdisciplinary working. Participants voiced a need for interdisciplinary training on frailty, and frailty tools that facilitate peer-learning, a shared understanding of frailty, and consistent assessment of frailty within and across specialities.

**Conclusions:**

These findings underscore the need to: (i) move beyond biomedical descriptions of frailty; (ii) further explore the interacting nature of the various components of the frailty umbrella, particularly the role of modifiable factors such as psychological and socioeconomic resilience; (iii) care for frail older adults using holistic, interdisciplinary approaches; and (iv) promote interdisciplinary training around frailty and frailty tools to facilitate a shared understanding and consistent assessment of frailty within and across specialities.

**Electronic supplementary material:**

The online version of this article (10.1186/s12877-019-1069-3) contains supplementary material, which is available to authorized users.

## Background

Frailty is a condition of vulnerability characterised by a loss of biological reserves across a range of physiological systems and functional domains. Older adults with frailty are at an increased risk of adverse outcomes such as disability, hospitalisation, nursing home admission and mortality [1, 2]. Assessment of frailty enables care providers to identify and anticipate the multidimensional needs of older adults, and tailor care to avert harm and improve outcomes [3].

Despite the vast amount of available research on the identification of frailty, little is known about how different healthcare professionals view frailty in practice [4, 5]. Nor is there consensus about whether they have a shared understanding of frailty that ensures consistent assessment of older adults, properly informs planning across care settings, and facilitates collaboration among health care disciplines – a key component in frailty management [5–10]. A few studies have focused on the understanding of frailty among hospital staff. But frailty is seen across all health care settings and acute care facilities are neither typical, nor ideal places to care for frail older adults [11], especially in light of their well-documented preference for remaining at home [12–14].

This study aimed to fill this gap by exploring how community care staff from various specialties viewed frailty and whether they had a shared understanding and assessment approach to frailty. It adds to the growing body of knowledge around frailty by identifying the knowledge gaps and training needs of community care staff. The findings of this study can contribute to the development of training programmes aimed at better identification and management of frailty among community dwelling older adults.

## Methods

### Design

Qualitative face-to-face, in-depth interviews were conducted with community care staff from various specialties. Braun and Clarks’ six phases of thematic analysis were used to guide the analysis of interview data. These six phases which begin with familiarising oneself with the data, and end with report writing, provide guidelines to help researchers conduct rigorous thematic analysis [15].

### Setting

Community and mental health services in Cambridgeshire and Peterborough (UK) are provided by 14 Neighbourhood Teams (NTs) each composed of approximately 30 members of various specialities including: community matrons, nurses and healthcare assistants; community psychiatric nurses and mental health support workers; physiotherapists, occupational therapists and therapy assistants. NTs work closely with community geriatricians, General Practitioners (GPs) and social workers to deliver integrated and holistic care to older adults living in the community [16]. Community and mental health services in Cambridge city are provided by four NTs. One of these four NTs was excluded from this study because the community matron, Maria Martin (MM) involved in recruiting and interviewing participants worked very closely with members of this NT. Consequently, another NT from one of the villages surrounding Cambridge was chosen as the fourth NT in this study.

### Recruitment strategy

Participants were recruited from four NTs in Cambridgeshire by MM who contacted managers of each neighbourhood team and gained permission to present the study and distribute the study flyer at neighbourhood team meetings. Potential participants who voiced an interest in taking part in the study during the team meetings or who contacted MM after these meetings were sent a copy of the participant information sheet via email. MM liaised with potential participants to schedule a convenient time and place to conduct interviews.

### Data collection

Three members of the research team (JC, MM & RS) conducted face-to-face, semi-structured interviews using an interview guide (Additional file 1). The interview guide was developed to elicit responses about how frailty was viewed and included questions about participants’ current role in the community; what frailty meant to them; whether they thought their colleagues viewed frailty in a similar manner to them; and how they assessed frailty in everyday practice. The interview guide was reviewed by members of the Frailty Trajectories Patient and Public Involvement Group and was piloted on community care staff from one of the neighbourhood teams excluded from the study. Written consent was sought by interviewers prior to the commencement of interviews. Audio recorded interviews ranging from 30 to 90 min were held in meeting rooms across four NTs between October and December 2017.

### Data analysis

All interview recordings were transcribed verbatim, anonymised and coded using the NVivo software package (Version 11). Interview transcripts were re-read by two members of the research team (JC & RS) and independently coded using thematic analysis to identify similarities and differences within and between themes across the different community care specialties. Codes were developed deductively using topics covered in the interview guide (e.g. description of frailty) and inductively based on codes that emerged during the analysis of the interviews (e.g. working together). Codes were assigned, agreed upon, collated and arranged into themes by JC and RS.

## Results

**Fig. 1:**
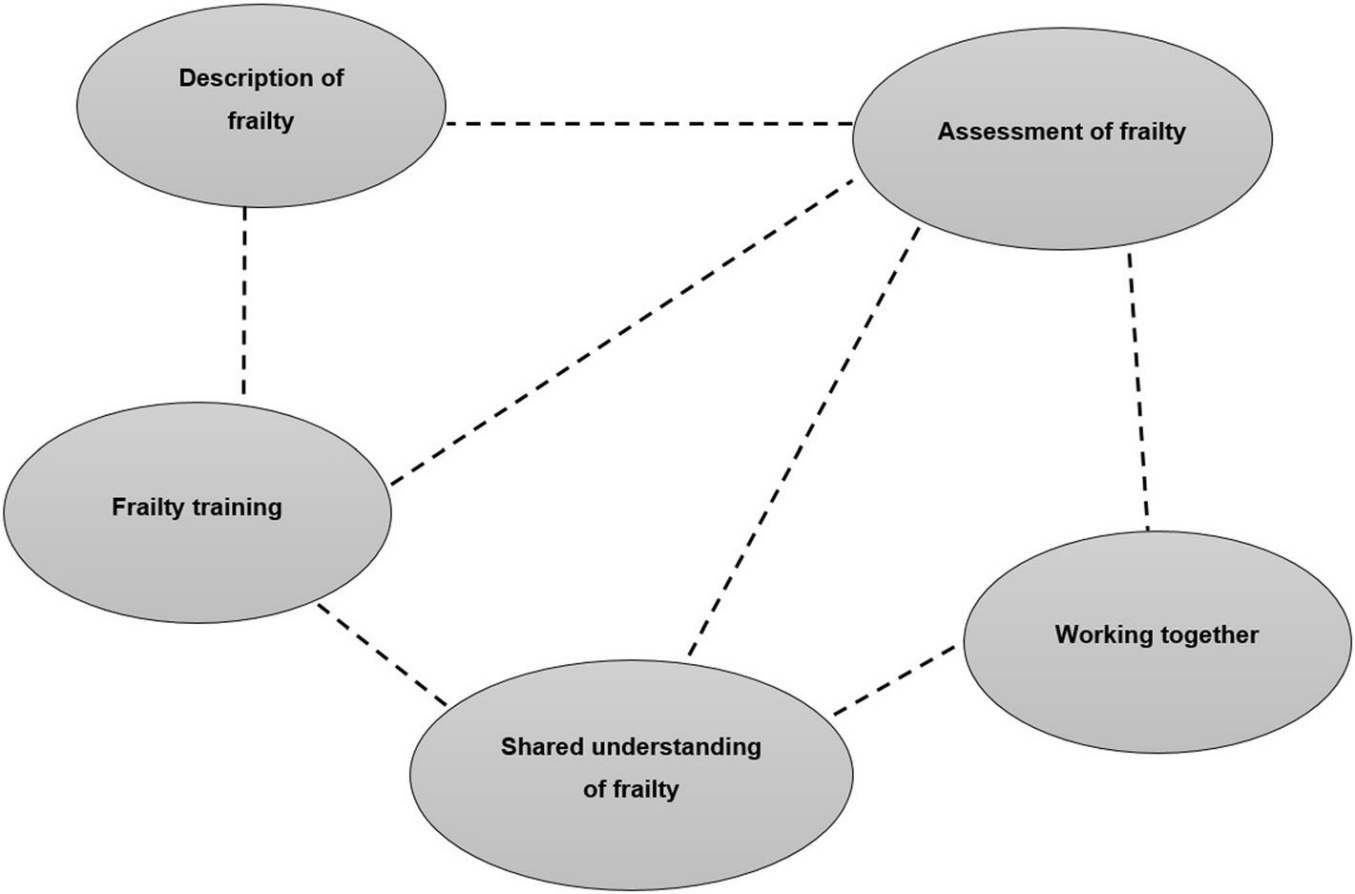
Thematic map

A total of 22 participants from seven different specialities were recruited from four NTs across Cambridgeshire. The characteristics of the participants are presented in Table 1. The names of NTs have not been included to ensure participant anonymity. As shown in Fig. 1, five main themes emerged from the analysis of participant interviews, namely: (i) description of frailty; (ii) shared understanding of frailty; (iii) assessment of frailty; (iv) working together; and (v) frailty training. Each theme and subsequent subthemes are described below alongside relevant interview extracts. Quotations are attributed by participant group and unique participant number. Unless otherwise stated, it should be assumed that the findings presented were voiced across all specialities i.e. by at least one member of all seven community care specialities.

**Table 1:** Characteristics of study participants

Participant characteristics	Number of participants
Number of participants	22
Speciality	
Healthcare assistant (HCA)	2
Nurse (N)	5 (3 Band 5^a^; 2 Band 6 ^b^)
Occupational therapist (OT)	4
Physiotherapist (PT)	4
Psychiatric nurse (CPN)	3
Social worker (SW)	2
Therapy assistant (TA)	2
Neighbourhood teams (NTs)	
NT1	7 (1HCA, 3 N, 2PT, 1SW)
NT2	5 (1 N, 1CPN, 2OT, 1SW)
NT3	6 (1HCA, 1TA, 2OT, 1PT, 1SW)
NT4	4 (1 N, 1TA, 1OT, 1PT)
Years of experience	5 months – 20 years
Gender	
Male	1
Female	21

^a^ staff nurses, ^b^senior staff nurses. *HCA* healthcare assistant, *N* nurse, *OT* occupational therapist, *PT* physiotherapist, *CPN* community psychiatric nurse, *SW* social worker, *TA* therapy assistant

### Theme 1: Description of frailty

**Fig. 2:**
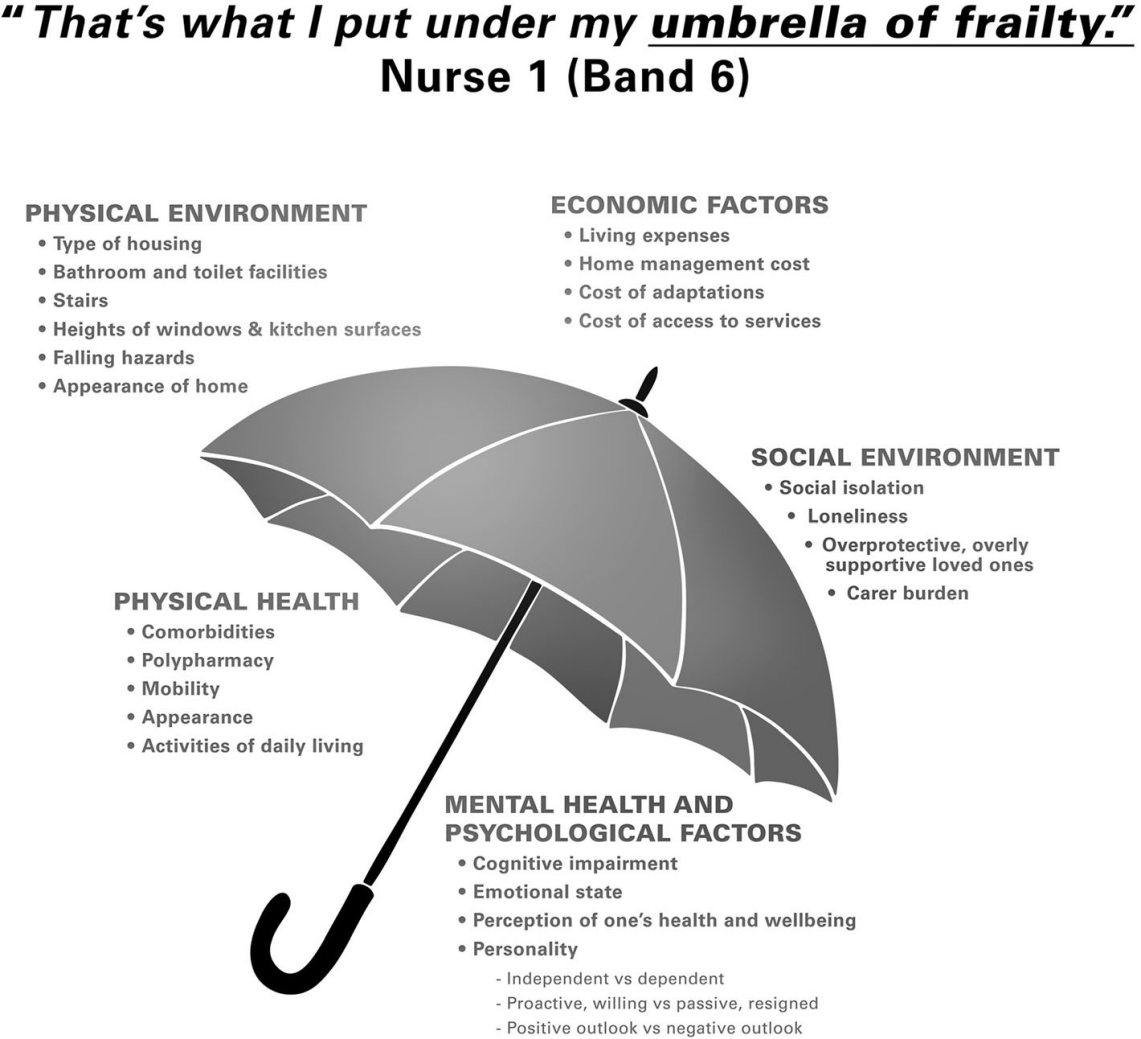
Components of the frailty umbrella

There was a general consensus among participants of all specialities that although frailty was associated with increasing age, it was not a requisite of ageing. Frailty was described as a very general word which was often difficult to define and had a different meaning among community care staff than in the lay world. To members of the lay world, it was thought to carry a negative connotation potentially implying end of life. Among community care staff of various specialities, frailty was described as an “*umbrella”* that encompassed: physical health, mental health and psychological factors, social factors, physical environment, and economic factors as shown in Fig. 2. These components were described as interacting factors i.e. they influenced and were influenced by other components of the frailty umbrella and increased the vulnerability of older adults to negative outcomes such as hospital admission and falls. Some specialties differed in the negative outcomes they cited. For example, psychiatric nurses emphasised risk of suicide, nurses mentioned risk of infection and pressure sores, and therapy staff i.e. occupational therapists and physiotherapists discussed risks associated with mobility.

*“they’ve got lots of complex factors that are all interacting, that they need more of a MDT approach”* PT2

*“In the social work role, you deal with the physical, the mental health, the social and the relationships aspect and the financial and, increasingly, the housing aspect as well, and so obviously, you know, we see frailty especially in kind of many forms.”* SW1

The various components of the frailty umbrella appeared to interact and produce a dynamic state. Numerous accounts were given of acute episodes of poor physical health or mental health and psychological factors; changes in physical environment; and social circumstances such as a bereavement which gave rise to temporary changes in the appearance of frailty. This was often described as good and bad days, time of day, or seasons where older adults appeared frailer than usual, as illustrated in the quotation below.

*“he can present as frail but not all the time, when he’s physically unwell he’s quite vulnerable but not all the time,”* CPN2

In the sub-themes below, the various components of the frailty umbrella will be presented. A description will be given of how the different components were individually perceived as indicators of frailty. This will then be followed by accounts of how each component influenced and was itself influenced by the other components of the frailty umbrella; highlighting the interactive nature of the various components of the frailty umbrella.

### Subtheme 1: Physical health

Participants described the physical appearance of frailty, a range of physical comorbidities, and polypharmacy. However, across all specialities, mobility took centre stage in discussions of physical frailty. For example, accounts were given of how conditions such as diabetes, arthritis and chronic obstructive pulmonary disease hindered mobility. Discussions about the appearance of frailty related to descriptions of stature, skin integrity, cleanliness and mobility. Words such as delicate, underweight, unkempt, weak, bruised, bent-over, pale and tottering were given as common features of the visual appearance of frailty.

*“So yeah, someone who’s probably unsteady on their feet … someone that appears vulnerable, requires assistance to complete normal day-to-day things, yeah.”* PT1

In addition to its role in physical frailty, across all specialities, mobility was said to interact with various other elements of the frailty umbrella and was frequently cited as a key factor that either facilitated or hindered the ability of older adults to engage in the social world around them. Mobility was thought to deeply affect the ability to carry out everyday activities such as toileting, bathing, food preparation etc. Difficulty or inability to *“manage”* or *“cope”* with everyday activities was deemed to be an indicator of frailty. The loss of independence and/or inability to carry out these activities was said to influence mental health and worsen frailty by lowering mood and self-esteem, increasing anxiety about ones’ health state and ability to cope, and creating a sense of helplessness and depression.

*“I think frailty is generally referred to someone's mobility more than anything else”* N5

### Subtheme 2: Mental health and psychological factors

Chronic mental health problems were described across all specialities as indicators of frailty and were often thought to cause older adults to appear frail. Furthermore, differences in personality and perceptions of one’s health and wellbeing were cited as contributors to frailty. Some older adults were described as seeming comfortable in the sick role, perceiving themselves as frail and being receptive or demanding of care. Others were said to come across as *“fiercely independent”*, rejected the sick role, perceived themselves as a burden and as such refused care. Accounts of both outlooks were described as positively and negatively influencing frailty. Positive in the sense that those who accepted the sick role sought care, albeit to the point that they became too dependent on others. Those who rejected the sick role, proactively tried to improve their health status and their ability to remain independent, yet refused care to their detriment.

Mental health and psychological factors were thought to influence other facets of the frailty umbrella. For example, memory loss, dementia and Alzheimer’s disease were said to negatively influence financial management, medication taking, everyday activities such as toileting and food preparation, perception of danger, and awareness of ones’ surroundings, as illustrated in the quotation below by a healthcare assistant. Furthermore, physiotherapists cited cognitive impairment as a hindrance to the understanding of and participation in rehabilitation activities which influence the physical health aspects of frailty.

*“things like making themselves a boiled egg but leaving the oven on and almost setting fire to the place, which has happened on numerous occasions, tripping the electric and not knowing that it’s gone and they’re sitting there in the cold.”* HCA2

Across all specialities, mental health and psychological factors were also thought to be influenced by the other aspects of frailty, highlighting the interactive nature of the frailty umbrella. For example, the loss of independence and social engagement that can accompany frailty, in some instances, was said to foster a negative emotional state that can cause older adults to appear frailer. It also hindered the willingness to seek help, carry out everyday activities and engage in rehabilitation activities. This in turn had a negative influence on the physical health aspects of frailty. Physiotherapists emphasised that such older adults require a lot of encouragement. In contrast, across all specialities, older people who had a positive, motivated and proactive outlook were perceived as less frail despite apparent and in some cases significant physical frailty as illustrated in the quotation below by a therapy assistant.

*“if she has a difficulty with something or a question she will ring me, she’s quite proactive in that sense so that almost can sometimes counteract what I’m seeing in front of me sometimes, you know, she’s not a frail person in herself always but physically she does have a lot of weakness”.* TA1

### Subtheme 3: Social environment

The absence or lack of the involvement of family, friends or neighbours who could act as informal carers and help older adults safely complete their rehabilitation activities and everyday activities, provide care, and aid early detection of worsening health, was thought to both highlight frailty and contribute to worsening frailty. Many participants across all specialities mentioned that their assessment of frailty included discussions and observations about the presence or absence of family and friends to provide support for older adults. A few participants confirmed that they would deem older people who lacked a support system as frailer than their counterparts who did have support from family or friends. It should however be noted that the presence of a social support network was not always deemed to be beneficial for frail older adults. Some families were described as overprotective, overbearing and overly supportive, which could create a sense of dependence and hinder the willingness or ability of older adults to care for themselves potentially contributing to worsening frailty. Although less common, the negative impact of frail adults having to care for other frail family members, particularly in relation to frail married couples, was also discussed by all specialities except therapy and healthcare assistants. In the quotation below, a nurse recounts a home visit where she became worried for a wife who was caring for her frail husband.

*“he’s sitting on the loo with his wife trying to help him and he’s struggling to get up off that toilet and back, walk anywhere. I mean that is total frailty in my mind. He no longer can cope with everyday living without support. But then I saw his wife as frail and she’s not on my caseload but she is frail and I just thought she’s going to not be able to cope”* N3

*“Or if you have an elderly couple who have both got problems and again there’s no family or there’s difficult family relationships then again they become very at risk, very frail.”* OT2

The prevailing narrative across all specialities about the interactions between social environment and the other aspects of the frailty umbrella was that social isolation often fostered a negative emotional state and outlook which made communicating and engaging with older adults difficult. As previously described under the mental health and psychological factors subtheme, a negative emotional state could influence one’s willingness to engage with care and thus impacts the physical health aspects of frailty. However, it was mentioned by therapy staff that isolated older adults can sometimes be more engaged – though not necessarily more adherent to rehabilitation – because they are grateful for the company and want care visits to continue. This is described in the quotation below by a therapy assistant. Instances were also described of physical health impeding frail older adults from accessing and interacting with the physical environment and social world around them, creating according to one of the occupational therapists a *“hidden group of frail, vulnerable people who are not in society”*.

*“they live on their own and they like the company they might want to participate more because they know you might be going back to see them but also that doesn’t necessarily mean they’ll be compliant”* TA1

### Subtheme 4: Physical environment

Across all specialities, the living environment or place of residence of older adults was thought to highlight frailty and reflect their physical health and economic limitations in terms of their ability to clean, care for, maintain, and run their homes. For example, an accumulation of letters at the door was described as an indicator of being unable to reach the floor and pick up objects with ease. Poor heating and lighting was described across specialities as a potential sign of economic limitations. In the quotation below, an occupational therapist recounts how some of these signs of neglect can sometimes be used to highlight frailty prior to entering the home of older adults living with frailty.

*“so you normally see the garden outside before you walk in and you can go 'that's the house I'm going to! if they were on their own so there's normally a neglect there, you can see, you know like they're not able to cope, so you, when you go into the house if it's cold … they generally don't put on lights because they're worried about money.”* OT1

Across all specialities, physical environment was also described as a factor that contributed to frailty by influencing other elements of the frailty umbrella. For example, the height of windows and kitchen surfaces; stairs; bathing and toileting facilities; heating and lighting; the presence of falling hazards such as rugs were described as key elements of one’s physical environment that potentially contribute to physical frailty by restricting mobility, increasing the likelihood of falls and hindering the ability to carry out everyday activities.

Community care staff across all specialities recounted how mental health and psychological factors such as dementia and hoarding influence the physical environment of older adults. Whether that be in terms of forgetting to turn the heating on and sitting in the cold or accumulating items around the home which restrict mobility and increase the risks of falls. All of these contribute to worsening physical frailty, further demonstrating the interactive nature of the various components of the frailty umbrella.

*“you get a hoarder where, you know. We’ve got one lady who can’t use her bathroom, can’t use her toilet because she can’t get to them. So those sort of things, she’s at high risk of falls … if she could get through to her bathroom, she could walk through but of course her frailty then becomes worse because she’s not mobilising so she gets weaker”* CPN3

### Subtheme 5: Economic factors

The financial limitations and implications of accessing certain services and to a lesser extent, adaptations for both frail older adults and their carers’ were described by social workers and nurses as factors that highlighted and contributed to frailty and carer burden. They explained that concerns about finances were cited as a factor that brought stress and anxiety to older adults and to their informal carers. Economic factors were thought to influence the suitability of the living environment of frail older adults. With the exception of physiotherapists and healthcare assistants, all other specialities mentioned that the cost of rent, heating, maintaining a home – and in some instances the costs of home adaptations – could influence the physical environment as well as physical health elements of the frailty umbrella. This further demonstrates the interactive nature of the various components of the frailty umbrella.

*“There's an awful lot of problems with clients where, you know, wife used to manage finances and no longer can't, and they've got in a real mess and then that, like a house of cards, you know, whatever the analogy is, has impacted on everything else, their stress levels, their ability to self-care, their level of stress, the ability to sleep, which then affects their physical health, and it just rolls and rolls and you have to find a way to give a gap, give a space for them to think.”* SW1

### Theme 2: Shared understanding of frailty among care staff

Responses from participants across all specialities when they were specifically asked whether they felt their colleagues viewed frailty in a similar manner were mixed. Some believed that frailty was perceived similarly, with only slight differences within and across specialities. Others, particularly nurses, occupational therapists and physiotherapists, felt there was no shared understanding of frailty. Interdisciplinary training about frailty and frailty tools, MDT meetings, and standardised guidelines were cited as factors that could facilitate a shared understanding of frailty across care staff. Such an understanding was said to be hampered by the dynamic nature of frailty and the use of different frailty tools. Differences in personality, experience, task focus and specialisation were also cited as factors that hindered the existence of a shared understanding of frailty. For example, nurses were thought to focus on the medical aspects of frailty, occupational therapists on functional abilities, physiotherapists on mobility, and psychiatric nurses on mental health. Furthermore, social workers felt there was no or poor understanding among other specialities about the social aspects of frailty. Likewise, psychiatric nurses felt the same about mental health and psychological factors i.e. shared understanding across other specialities was poor. Nevertheless, the descriptions of frailty voiced by participants in this study demonstrated that across all specialities, they shared the narrative that frailty is an umbrella term of interacting factors, namely: physical health, mental health and psychological factors, social factors, physical environment, and economic factors.

*“I think they [therapy staff] have the same understanding, if not more about what somebody can do for theirselves, walking and getting things because their whole job is re-enable them, get them to do as much as they can for theirselves”* N3

### Theme 3: Assessment of frailty

In accordance with the holistic picture represented by the frailty umbrella, there was a consensus across all specialities that the assessment of frailty requires a holistic approach. In the quotation below, for instance, a social worker warns that the failure to address any aspect of frailty could have a detrimental effect on the older person as a whole.

*“someone will do a really good piece about A, and there won't be a great response about B, … … . but that bit’s not dealt with. Because that bit’s not dealt with, it pulls everything else down”* SW1

Participants across all specialities explained that frailty assessment begins from the minute they arrive at the home of an older adult and includes observing and asking questions about the various components of the frailty umbrella in order to gain a holistic picture of the older adult and their care needs. With the exception of social care workers, therapy assistants and healthcare assistants, all other specialties mentioned that in addition to observing and asking questions, they used various tools which they believed indirectly assessed frailty. Nurses described using the initial holistic assessment tool, a mental health checklist and Waterlow score to assess risk of pressure sores [17]; occupational therapists mentioned that they used an initial holistic occupational therapy tool but like physiotherapists also used the EuroQol to assess quality of life [18] and the Community Dependency Index to assess independence in carrying out self-care activities [19]. In addition to these tools, physiotherapists recounted using the Berg Balance Scale to measure balance [20] and the Tinetti assessment tool to assess gait and balance [21]. It should be noted that the specialities that mentioned using additional tools were the specialities who believed there was no shared understanding about frailty. Furthermore, the differences in the tools mentioned mirror the perceived speciality bias to frailty i.e. nurses having a more medical emphasis and therapy staff an emphasis on functional abilities and mobility. This is consistent with the different emphasis in the negative effects of frailty previously discussed in the description of frailty theme. It also supports the belief among participants that the focus on their specialities hindered the existence of a shared understanding about frailty.

*“they’ll probably ask me sometimes if I’d like a cup of tea, I would often say yes, because it’s interesting to watch somebody when they go into their kitchen, we do a lot of assessment, on watching somebody going into their kitchen and whether they can fill a kettle, whether they can lift it, whether they have the strength to do that. Whether they can go to the fridge and get the milk, whether they know where things are in their kitchen. We can tell quite a lot from a very simple task of whether they know how to make a cup of tea or even where their kitchen is. So you know, so we’re assessing the whole time we’re there”* CPN 3

The revised 9-point Clinical Frailty Scale is currently used by some community care staff in NTs across Cambridgeshire and Peterborough as a means of identifying frailty and assessing the risks and needs of older adults living in the community [22]. With the exception of social workers who do not use the scale, across all other specialties, some mentioned that they had been asked to use the Clinical Frailty Scale and found it to be a quick and easy guide. However, many others across all specialities excluding social workers voiced barriers to its use, including lack of knowledge about the tool; lack of consistency in scoring by professionals; lack of integration between the physical and mental health components of the scale; and the dislike of putting people in restrictive or specific categories which they referred to as *“pigeon holes”.* When asked what an ideal frailty tool should be, many cited the following as preferred characteristics of a frailty tool: holistic assessment of the various components of the frailty umbrella; bullet points of key things to look out for on small cards that can be carried with ease; and most importantly, signposts or suggestions on what to do next to help the older adults in their care manage their frailty.

*“It [ideal frailty tool] would be one that, it would help you understand like different areas of frailty and maybe it would be able to point you in the right direction if you think, like if this, “If you think this is going to happen like maybe do that,” sort of thing so that, but yeah.”* N4

### Theme 4: Working together

Across all specialities, holistic assessment of frailty was thought to be facilitated by working with colleagues of various specialities within NTs. The extent of joint working appeared to vary across specialties and NTs with some claiming that the formation of NTs did facilitate access to colleagues of various specialities, while others felt that more could be done to encourage joint working and integration. The nature of joint working was described across specialties as taking two main forms, namely: (i) joint visits and multidisciplinary team (MDT) meetings and (ii) referrals and information sharing. Joint visits were described mainly by nurses, occupational therapists and physiotherapists as efficient, productive and beneficial for the care of older adults living with frailty, particularly for complex cases where additional input was needed. Similarly, MDT meetings were described as an avenue to discuss complex cases, elicit input from various specialities, and ensure that the team was aware of available resources and working in a consistent, harmonious manner. This was the case across all specialities with the exception of therapy assistants and healthcare assistants who did not attend these meetings. The attendees, frequency and logistics of these meetings varied across NTs. However, concerns were raised that busy schedules and limited staffing meant that such meetings were often poorly attended and lacked the range of necessary specialisations.

Sharing information about older adults on their caseload face-to-face and via computer records was deemed as crucial. It ensured that community care staff arrived at home visits equipped with adequate and accurate information. They often referred to instances where this was not the case as attending home visits *“blind”*. The use of shared computer records was perceived by nurses, psychiatric nurses, occupational therapists and physiotherapists to be essential to allow access to further information about older adults living with frailty to ensure they had a better picture or understanding of them and their potential needs prior to home visits. With the exception of therapy assistants and healthcare assistants, co-location in the same building was also thought to improve information sharing and joint working because it alleviated issues caused by the use of different computer systems and facilitated quick, face-to-face communication and arrangement of referrals and joint visits; cohesion (by lowering professional boundaries); and the development of a holistic picture of older adults living with frailty and their care needs.

*“ … ..there is nearly nothing so negative as to draw apart, and there's nothing nearly so positive as to put people together, because that weird unknown thing that you just do a reform for and refer to, suddenly becomes people who can explain themselves, and that inter-colleague dialogue happens a thousand times more when you are sitting side by side or room by room or whatever it might be.”* SW1

### Theme 5: Frailty training

The majority of participants across all specialities expressed a desire for more training on frailty and the use of frailty tools to improve their knowledge and understanding of frailty and aid their assessment of frailty among older adults living in the community. Common to all specialties was the desire for frailty training to clarify the purpose of using the Clinical Frailty Scale, detailing: what the score was being used for and the benefits and impact to both staff and older adults of using the Clinical Frailty Scale. Furthermore, with the exception of therapy assistants, participants across all specialties emphasized that frailty training needed to provide information to ensure that scoring using the Clinical Frailty Scale was consistent across specialties.

*“So why, why the need for the frailty score is there because I think a lot of people don’t quite understand because we see, we see different frailties and we act on it already so why would this new thing that’s come about? Maybe some sort of unification of this, how we’re scoring. Yeah, no, I think probably why and how would be the most important things, you know, so there is, you know, there is room for merging a couple of them, can you do a seven stroke eight, can you do a two stroke three because some people don’t fit in exact boxes”* HCA2

There was a general consensus across specialities and across participants that the ideal way to receive frailty training was face-to-face, in an environment that would encourage discussion, questions, and thus facilitate peer learning across a range of care specialties i.e. an interdisciplinary training experience. This was thought to encourage learning and facilitate a shared understanding of frailty within and across specialities. Participants also highlighted the need for a practical learning session, potentially involving case studies, led by staff dealing with frailty on a daily basis. They emphasised the need for training that was of practical benefit to their everyday practice rather than a theoretical learning session where they had to sit and listen and to someone talk at them. A healthcare assistant described this as “*death by Powerpoint”.* The suggested avenues for face-to-face training sessions included inductions and multidisciplinary team meetings where as many specialties as possible were present.

*“So, I don’t know how achievable that is, but I think if that could be used, like in neighbourhood meetings can be used to just have a generalised discussion, not someone telling you what frailty is, but instead having professionals discussing about frailty themselves … .It is a part of peer learning, definitely it is peer learning, because you can hear lots and lots of things, you can do lots and lots of online training, but it’s different when you go into the field and when you practice it, because theory is there, which you need to support your practical knowledge. But there is an experience of different people, which comes from peer learning, yes.”* PT4

## Discussion

This study aimed to explore how community care staff of various specialties viewed frailty and whether they had a shared understanding and assessment approach to frailty. The findings demonstrated that despite describing frailty as difficult to define, participants across all specialities viewed it as an umbrella term that consisted of interacting physical, mental health and psychological, social, environmental, and economic factors. These findings are in accordance with emerging literature that highlights the need to move beyond the traditional biomedical description of frailty [5, 9, 23, 24]. It should however be noted that whilst the role of mental health, psychological factors and social factors are now recognised, the influence of physical environment and economic factors are less recognised but have been documented by a few studies [25–27]. This may be because research on frailty in older adults living in the community where these factors i.e. physical environment and economic factors would be more apparent is sparse.

This study found that each of the various components of the frailty umbrella were individually viewed as indicators of frailty, but also interacted with each other in a complex manner. This is in agreement with Rockwood’s “dynamic model of frailty” which highlights the complex interplay within and between various components of frailty [28]. This study exploring community care staff views of frailty did not aim to unpick the nature of these complex interactions. However, it was apparent in the findings that certain elements of the frailty umbrella such as: personality differences; perceptions of one’s health; and the presence of family and friends who could to provide informal care, had the potential to either aid or hinder the management of frailty among older adults living in the community. Psychological resilience, and to a lesser extent socioeconomic resilience, have been identified as key factors that aid the management of frailty [9, 12, 27, 29, 30]. These factors are under-researched, modifiable and pending further research, could help healthcare professionals identify and mitigate negative coping strategies or responses to frailty [29, 30]; especially in older adults who are refusing care or who are not adherent to their rehabilitation activities.

Mobility was found to be a central tenet of frailty in this study and in a study exploring the experience and assessment of frailty by six community nurses [11]. This is of little surprise as mobility indicates complex system failure in accordance with the pathophysiology of frailty and its influence on other elements of the frailty umbrella, specifically: functional ability, loss of confidence and social withdrawal is well-documented [31]. Nevertheless, a study exploring the views of frailty among healthcare professionals in Sweden did not find mobility to be a key characteristic of frailty [5]. This may be because participants in the Swedish study were hospital based staff who may have assessed their patients in the hospital. It is well-documented that levels of physical activity among older adults in hospitals is low [32]. A Cambridge based study found that 24 participants spent only 1.1% of their time in hospital standing and moving [33].

There was no consensus among participants in this study about whether a shared understanding of frailty existed among themselves and their colleagues. Yet their narratives demonstrated that they did in fact have a shared understanding that frailty was an umbrella term of interacting factors. Furthermore, the narrative of participants in this study highlights that although they had a general shared understanding of the complex, multifactorial nature of frailty, there were some differences across specialities in their key areas of emphasis. Similarly, Gustafsson’s study found the same shared narrative and difference in emphasis among Swedish health professionals of various specialites [5]. The findings of both these studies contradict the findings of a quantitative study which concluded that there was no shared understanding of frailty among hospital based healthcare professionals [7]. However, the methodology of this study differed and agreement of only 3 healthcare professionals, a nurse, a resident and a chief resident was assessed. Partcipants in the current study voiced their beliefs that an increase in joint working across disciplines and interdisciplinary training in a face-to-face interactive format that encourages peer learning within and across specialities would faciliate a much needed shared understanding of frailty.

In agreement with existing literature and the holistic description of frailty, the assessment of frailty in this study was thought to require a holistic approach facilitated by interdisciplinary working [8, 10]. This was the case across all specialities included in this study. Indeed, interdisciplinary working and training around frailty and frailty tools, which participants in this study and in Britton’s study [11] cited as an area where more knowledge and training was needed, may (i) facilitate an awareness of the extent and limitations of the narrative around frailty shared by community care staff; (ii) encourage joint-working; and (iii) facilitate consistency in the assessment and management of frailty among community dwelling older adults.

### Strengths and limitations

All interviewers agreed that data saturation had been reached on completion of 22 interviews as no new themes emerged [34]. However, no community matrons (Band 7 nurses), community geriatricians or GPs were included in this study. These healthcare professionals also provide care for frail older adults living in the community and work alongside the community care staff interviewed in this study. Consequently, their narrative would have helped to paint a more complete picture of the views of frailty among healthcare professional who care for frail older adults living in the community. Furthermore, participants were a self-selected group who may have had an increased interest in frailty. Consequently, their views may not be representative of all community care staff. Although reliability of the data was enhanced by the independent coding and agreement of codes by a community care and an academic interviewer [35], participants were aware of the professional roles of their interviewers. Consequently, they may have assumed shared knowledge with community care interviewers and thus provided less information about some areas, yet expanded on others. The reverse may have been the case for the academic interviewer. To minimise this, the same interview guide was used by all interviewers. Furthermore, throughout the research process, all of the researchers engaged in reflexivity, considering and discussing their own views of frailty and the impact of these views and their academic and clinical backgrounds on the research process [34].

## Conclusions

There was a general consensus that frailty was a multifaceted, dynamic, umbrella term that encompasses interacting physical, mental health and psychological, social, environmental, and economic factors. Frailty was thought to require a holistic assessment and management approach facilitated by interdisciplinary working. However, healthcare professionals of different specialities emphasised the role of certain areas of the frailty umbrella in their narratives of the description and assessment of frailty in older adults living in the community. The narrative of nurses had a more medical focus, therapy focused specialities discussed functional abilities and mobility more, and the narrative of psychiatric nurses and social workers placed more emphasis on mental health and social factors respectively.

Improving our understanding of psychological and socioeconomic resilience is necessary to enable healthcare professionals to identify and respond to negative coping strategies and responses in an interdisciplinary manner to improve the outcomes and experiences of community dwelling frail older adults.

There is a need for greater interaction and collaboration between and among healthcare professionals of various specialities – whether that be as a result of being co-located, MDT teams, or interdisciplinary training sessions. This is key to facilitate a shared understanding of frailty and aid its assessment and management. Furthermore, more training is needed around frailty and the various frailty tools. Such training needs to be practical in nature, reflecting the reality of everyday practice. It should aim to increase knowledge and understanding of frailty and the use of various frailty tools among healthcare staff, and promote consistent practice in the hope of improving the care of frail older adults living in the community.

## Additional file

Additional file 1: Interview guide. This document contains the list of questions and prompts used by interviewers to elicit responses about how study participants viewed frailty and assessed frailty in their everyday practice. (PDF 271 kb)

## Abbreviations

CPFT: Cambridge and Peterborough Foundation Trust
CPN: Community psychiatric nurse
GP: General Practitioners
HCA: Healthcare assistant
MDT: Multidisciplinary team
N: Nurse
NIHR: National Institute for Health Research
NT: Neighbourhood teams
OT: Occupational therapist
PT: Physiotherapist
SW: Social worker
TA: Therapy assistant
UK: United Kingdom
