# Supplementary material for: Frailty: an in-depth qualitative study exploring the views of community care staff
Source: BMC Geriatr. 2019 Feb 19;19:47. doi: 10.1186/s12877-019-1069-3 (PMC6381739; doi:10.1186/s12877-019-1069-3)
Supplement: Supplementary file 1 — Interview guide. This document contains the list of questions and prompts used by interviewers to elicit responses about how study participants viewed frailty and assessed frailty in their everyday practice. (PDF 271 kb) [file 12877_2019_1069_MOESM1_ESM.pdf]

Interview guide

Introduction

Aim to introduce self and study using information sheet:

- Introduce self
- Introduce study
- Purpose of study
- Freedom and process of withdrawing from the study
- Duration of interview
- Recording of interview
- Confidentiality and anonymity
- Obtain written consent to proceed

RECORDER ON

Aim: explore to which community staff group the interviewee belongs and their clinical background and experience.

1. **Please tell me about yourself, within your current role in the community**

   Prompts:

   i. What qualifications do you have?
   ii. How long have you worked in the community?
   iii. How long in total have you been practising as a ---?

Aim: explore how interviewee views frailty.

2. **What does frailty mean to you?**
3. **Can you please describe a frail patient that you have seen in your everyday practice?**

Aim: explore how community care staff of various specialities go about assessing frailty.

4. **Can you please tell me how you go about assessing frailty?**

   Prompt:

   Do you know any frailty tools?

5. **Do you think the other people you work with view frailty in a similar way?**

Aim: explore how community care staff of various specialties view the frailty tools

6. **Have you used any frailty tools and how did you find them?**

7. **Do you have a preferred frailty tool?**

   Consider response that no tool is required

   Can you please tell me why you don't think we need a tool?

Aim: explore the elements of frailty that community care staff of various specialties wish to assess

8. **If I could ask you to describe your perfect frailty tool, what would it look like?**

Ending Interview

9. **Is there anything you would like to mention that hasn't been asked that you think is relevant, or anything you wish to elaborate on?**

RECORDER OFF

Thank interviewee for their time and the information that they have provided

Ask if they would like to know about the findings of the study, and provide a contact email address.
